# World Allergy Organization-McMaster University Guidelines for Allergic Disease Prevention (GLAD-P): Probiotics

**DOI:** 10.1186/s40413-015-0055-2

**Published:** 2015-01-27

**Authors:** Alessandro Fiocchi, Ruby Pawankar, Carlos Cuello-Garcia, Kangmo Ahn, Suleiman Al-Hammadi, Arnav Agarwal, Kirsten Beyer, Wesley Burks, Giorgio W Canonica, Motohiro Ebisawa, Shreyas Gandhi, Rose Kamenwa, Bee Wah Lee, Haiqi Li, Susan Prescott, John J Riva, Lanny Rosenwasser, Hugh Sampson, Michael Spigler, Luigi Terracciano, Andrea Vereda-Ortiz, Susan Waserman, Juan José Yepes-Nuñez, Jan L Brożek, Holger J Schünemann

**Affiliations:** Pediatric Hospital Bambino Gesù, Vatican City, Italy; Department of Pediatrics, Nippon Medical School, Tokyo, Japan; Department of Clinical Epidemiology and Biostatistics, McMaster University Health Sciences Centre, Room 2C19, 1200 Main Street West, Hamilton, ON L8N 3Z5 Canada; Tecnologico de Monterrey School of Medicine and Health Sciences, Monterrey, Mexico; Department of Pediatrics, Samsung Medical Center, Sungkyunkwan University School of Medicine, Seoul, South Korea; Department of Pediatrics, College of Medicine and Health Sciences, United Arab Emirates University, Al-Ain, United Arab Emirates; Faculty of Medicine, University of Toronto, Toronto, ON Canada; Charité Klinik für Pädiatrie, Berlin, Germany; Department of Pediatrics, University of North Carolina, Chapel Hill, NC USA; University of Genoa, IRCCS AOU San Martino-IST, Genoa, Italy; Department of Allergy, Clinical Research Center for Allergology and Rheumatology, Sagamihara National Hospital, Kanagawa, Japan; Department of Pediatrics and Child Health, Aga Khan University Hospital, Nairobi, Kenya; Department of Paediatrics, Yong Loo Lin School of Medicine, National University of Singapore, Singapore, Singapore; Department of Primary Child Care, Children’s Hospital, Chongqing Medical University, Chongqing, China; Department of Immunology, Perth Children’s Hospital, Telethon KIDS Institute, School of Paediatrics and Child Health, University of Western Australia, Perth, Australia; Department of Family Medicine, McMaster University, Hamilton, ON Canada; Allergy-Immunology Division, Children’s Mercy Hospital & University of Missouri – Kansas City School of Medicine, Kansas City, MO USA; Jaffe Food Allergy Institute, Mount Sinai School of Medicine, New York, NY USA; Food Allergy Research & Education (FARE), McLean, VA USA; Department of Child and Maternal Medicine, University of Milan Medical School at the Melloni Hospital, Milan, Italy; Department of Medicine, McMaster University, Hamilton, ON Canada; Allergology Department, Hospital Infantil Universitario Niño Jesus, Madrid, Spain

**Keywords:** Allergy, Prevention, Probiotics, Practice guidelines, GRADE

## Abstract

**Background:**

Prevalence of allergic diseases in infants, whose parents and siblings do not have allergy, is approximately 10% and reaches 20–30% in those with an allergic first-degree relative. Intestinal microbiota may modulate immunologic and inflammatory systemic responses and, thus, influence development of sensitization and allergy. Probiotics have been reported to modulate immune responses and their supplementation has been proposed as a preventive intervention.

**Objective:**

The World Allergy Organization (WAO) convened a guideline panel to develop evidence-based recommendations about the use of probiotics in the prevention of allergy.

**Methods:**

We identified the most relevant clinical questions and performed a systematic review of randomized controlled trials of probiotics for the prevention of allergy. We followed the Grading of Recommendations Assessment, Development and Evaluation (GRADE) approach to develop recommendations. We searched for and reviewed the evidence about health effects, patient values and preferences, and resource use (up to November 2014). We followed the GRADE evidence-to-decision framework to develop recommendations.

**Results:**

Currently available evidence does not indicate that probiotic supplementation reduces the risk of developing allergy in children. However, considering all critical outcomes in this context, the WAO guideline panel determined that there is a likely net benefit from using probiotics resulting primarily from prevention of eczema. The WAO guideline panel suggests: a) using probiotics in pregnant women at high risk for having an allergic child; b) using probiotics in women who breastfeed infants at high risk of developing allergy; and c) using probiotics in infants at high risk of developing allergy. All recommendations are conditional and supported by very low quality evidence.

**Conclusions:**

WAO recommendations about probiotic supplementation for prevention of allergy are intended to support parents, clinicians and other health care professionals in their decisions whether to use probiotics in pregnancy and during breastfeeding, and whether to give them to infants.

**Electronic supplementary material:**

The online version of this article (doi:10.1186/s40413-015-0055-2) contains supplementary material, which is available to authorized users.

## Executive summary

The purpose of this document is to provide evidence-based recommendations about the use of probiotic supplements for the primary prevention of allergies.

Prevalence of allergic diseases in infants is approximately 10% in those without an allergic parent or sibling and 20% to 30% in those with allergy in their relatives. Studies suggest that intestinal microbiota may modulate immunologic and inflammatory systemic responses and, thus, influence development of sensitization and allergy. Probiotics are living microorganisms that when administered to humans in adequate doses may confer a health benefit. They have been proposed to modulate immune responses and have been advocated as therapeutic and preventive interventions for allergic diseases.

### Methodology

The methods used to develop clinical recommendations in this document follow the Grading of Recommendations, Assessment, Development and Evaluation (GRADE) approach. The Guideline panel included clinicians and researchers in the field of allergy (allergists), pediatricians, primary care physicians, and methodologists. Potential conflicts of interests were managed as suggested by the World Health Organization.

The guideline panel developed and graded the recommendations and assessed the quality of the supporting evidence following the GRADE approach. The quality of evidence (also called confidence in the available estimates of health effects or certainty in the evidence) is categorized as: high, moderate, low or very low based on consideration of risk of bias, directness of evidence, consistency and precision of the estimates. Low and very low quality evidence indicates that the estimated effects of interventions are very uncertain and further research is very likely to influence resulting recommendations.

### Interpretation of strong and conditional recommendations

The strength of recommendations is expressed as either strong (guideline panel recommends…) or conditional (guideline panel suggests…) Understanding the interpretation of these two grades of strength of recommendations is essential for judicious health care decision-making and has explicit implications as follows:

#### Strong recommendation

- ◾ For patients: most individuals in this situation would want the recommended course of action, and only a small proportion would not.
- ◾ For clinicians: most individuals should receive the intervention. Adherence to this recommendation according to the guideline could be used as a quality criterion or performance indicator. Formal decision aids are not likely to be needed to help individuals make decisions consistent with their values and preferences.
- ◾ For policy makers: the recommendation can be adopted as policy in most situations.

#### Conditional recommendation

- ◾ For patients: the majority of individuals in this situation would want the suggested course of action, but many would not.
- ◾ For clinicians: recognize that different choices will be appropriate for individual patients and that you must help each patient arrive at a management decision consistent with his or her values and preferences. Decision aids may be useful in helping individuals to make decisions consistent with their values and preferences.
- ◾ For policy makers: policy-making will require substantial debate and involvement of various stakeholders.

### How to use these guidelines

The GLAD-P guidelines about the use of probiotics provide the basis for rational, informed decisions for clinicians, parents and other decision makers. Clinicians, patients, third-party payers, institutional review committees, other stakeholders, or the courts should never view these recommendations as dictates. No recommendation can take into account all of the often-compelling unique individual circumstances but provides guidance. However, no one charged with evaluating health care professional’s actions should attempt to apply the recommendations from these guidelines by rote or in a blanket fashion.

### Recommendations

Note: statements about the underlying values and preferences as well as qualifying remarks accompanying each recommendation are its integral parts and serve to facilitate more accurate interpretation; they should never be omitted when quoting or translating recommendations from these guidelines.

## Recommendation 1

The WAO guideline panel suggests using probiotics in pregnant women at high risk for allergy in their children, because considering all critical outcomes, there is a net benefit resulting primarily from prevention of eczema (conditional recommendation, very low quality evidence).

### Values and preferences

This recommendation places a relatively high value on prevention of eczema in children, and a relatively lower value on avoiding possible adverse effects.

### Explanations and other considerations

There is lack of evidence that probiotics prevent any other allergy. In most studies probiotics were used in the last 3 months of pregnancy. This recommendation applies to otherwise healthy women and cannot be generalized to those with compromised immune system function. High risk for allergy in a child is defined as biological parent or sibling with existing or history of allergic rhinitis, asthma, eczema, or food allergy.

## Recommendation 2

The WAO guideline panel suggests using probiotics in women who breastfeed infants at high risk of developing allergy, because considering all critical outcomes, there is a net benefit resulting primarily from prevention of eczema (conditional recommendation, very low quality evidence).

### Values and preferences

This recommendation places a relatively high value on prevention of development of eczema, and relatively lower value on avoiding possible adverse effects.

### Explanations and other considerations

There is lack of evidence that probiotics prevent any other allergy. This recommendation applies to otherwise healthy women and cannot be generalized to those with compromised immune system function. High risk for allergy in a child is defined as biological parent or sibling with existing or history of allergic rhinitis, asthma, eczema, or food allergy.

## Recommendation 3

The WAO guideline panel suggests using probiotics in infants at high risk of developing allergies, because considering all critical outcomes, there is a net benefit resulting primarily from prevention of eczema (conditional recommendation, very low quality evidence).

### Values and preferences

This recommendation places a relatively high value on prevention of eczema, and relatively lower value on avoiding possible adverse effects.

### Explanations and other considerations

There is lack of evidence that probiotics prevent any other allergy. This recommendation applies to otherwise healthy infants with the goal of preventing allergies. It does not apply to the use of probiotics for other indications, e.g. prevention of antibiotic-associated diarrhea or enterocolitis in premature infants.

## Scope and purpose

The purpose of this document is to evaluate the current evidence and provide guidance on the use of probiotics for the primary prevention of allergies. The target audience of these guidelines are general practitioners, pediatricians, specialists in allergic disease and immunology, respiratory medicine and dermatologists managing adults and children with any kind of allergy. General internists, and other health care professionals and policy makers involved in may also benefit from these guidelines. Policy makers interested in these guidelines include those involved in developing local, national or international plans with the goal to reduce incidence of allergy and resource direct and indirect costs related to allergic diseases [1]. This document may also serve as the basis for development and implementation of locally adapted guidelines.

## Introduction

Allergic diseases represent a spectrum of health conditions and a worldwide burden in different populations [2]. In infants, its prevalence depends highly on the allergic status of their parents, being approximately of 10% in those without an allergic parent or sibling, versus 20% to 30% in those with an atopic background in their first degree relatives [3].

In recent years, more attention has been given to the intestinal microbiota and its influence on sensitization and the origins of allergic disease, as it may modulate immunologic and inflammatory systemic responses [4]. The intestinal microbiota hypothesis has been proposed to explain the rising incidence of allergic disorders [5].

Probiotics are living microorganisms that when administered to humans in certain doses may confer a health benefit. They have been proposed as immune-modulators of the allergic response by affecting phagocytosis and production of pro-inflammatory cytokines, and thus are being advocated as therapeutic and preventive interventions for allergic diseases [6,7].

The Guidelines for Atopic Disease Prevention (GLAD-P) is a joint effort of the World Allergy Organization (WAO) and the Department of Clinical Epidemiology & Biostatistics at McMaster University to evaluate the current evidence on the preventive effect of probiotics, prebiotics, and vitamin D on allergic diseases and related clinically important outcomes. This document provides recommendations and the rationale for use of probiotics.

For clarity of communication we used the following definitions throughout the document:

***• Probiotics:*** "live microorganisms which, when administered in adequate amounts as part of food, confer a health benefit on the host” [8] Probiotics are present in everyday food (e.g. yoghurt or fermented milk) and they are a common exposure in almost everyone's life. For the purpose of this document we considered probiotics as a supplementary therapeutic agent used for the prevention of allergy; we have not considered what everyday general food people should consume. Of note, even though many species of microorganisms (e.g. Lactobacilli and Bifidobacteria) have been studied and the probiotic characteristics were confirmed for many of their strains, not all strains are probiotics.
***• High risk for allergy in a child***: biological parent or sibling with existing or history of allergic rhinitis, asthma, eczema, or food allergy [9].
***• Weaning or complementary feeding***: the period during which any other foods or liquids are provided along with breast milk or infant formula [10].

## Methods

### Panel composition and meetings

We followed the procedures and methodology using the guideline development checklist (GDC) [11] and the guideline development tool (GDT) [12] to assemble team of experts including allergists, pediatricians, family physicians and representatives of the general public.

The guideline panel included methodologist who helped preparing systematic reviews and evidence summaries following the GRADE (Grading of Recommendations, Assessment, Development and Evaluation) approach.

A face-to-face meeting was held in December 2013 coinciding with the WAO Symposium on Immunotherapy and Biologics in Chicago, Illinois. During the meeting the guideline panel discussed specific questions, the existing research evidence and made recommendations.

### Disclosure of potential conflicts of interest

Guideline panel members disclosed all potential conflicts of interest according to the World Health Organization policies. The chairs (AF, RP and HJS) reviewed and resolved all potential conflicts of interest of panel members (see Additional file 1 for the list of declared conflicts of interest for all panel members). During all deliberations, panel members with potential conflicts of interest abstained from decisions about specific questions being asked and recommendations related to their potential conflict of interest.

The WAO provided meeting facilities during its Symposium and financial support to perform systematic reviews to support recommendations. The views and interests of the WAO as well as of any commercial entity that provided external funding for WAO had no influence on the final recommendations.

### Formulating specific clinical questions and determining outcomes of interest

We used the electronic tools Guideline Development Tool (www.guidelinedevelopment.org) [12] and SurveyMonkey (www.surveymonkey.com) to brainstorm and subsequently prioritize questions related to the use of probiotics for the prevention of allergy.

The following questions were prioritized and addressed in this document:

1. Should probiotics versus no probiotics be used in pregnant women?

We intended to examine the effects in subpopulations of children at high and average risks for allergies (see definitions above).

2. Should probiotics versus no probiotics be used in breastfeeding women?

We intended to examine the effects in subpopulations of children at high and average risks for allergies (see definitions above).

3. Should probiotics versus no probiotics be used in infants?

We intended to examine the effects in subpopulations of children at high and average risks for allergies (see definitions above), exclusively and non-exclusively breastfed infants, and infants being weaned.

The guideline selected outcomes of interest for each question following the approach suggested by the GRADE Working Group [13]. All outcomes were identified a priori and the panel explicitly rated their relative importance for decision-making. Ranking outcomes by their relative importance can help to focus attention on those outcomes that are considered most important and help to resolve or clarify potential disagreements.

### Evidence review and development of clinical recommendations

Evidence summaries for each question were prepared by the methodologists (JB, CCG, JJYN and HJS) following the GRADE approach and using the Guideline Development Tool (www.guidelinedevelopment.org). All guideline panel members reviewed the summaries of evidence and made corrections when appropriate. We based the evidence summaries on a systematic review of the literature performed specifically for these guidelines (Cuello-Garcia et al. in preparation). We followed the methods of the Cochrane Collaboration (handbook.cochrane.org) and assessed the risk of bias at the outcome level using the Cochrane Collaboration’s risk of bias tool [14]. Subsequently, we assessed the quality of the body of evidence (i.e. confidence in the estimated effects) for each of the outcomes of interest following the GRADE approach based on the following criteria: risk of bias, precision, consistency and magnitude of the estimates of effects, directness of the evidence, risk of publications bias, presence of dose–effect relationship, and an assessment of the effect of residual, opposing confounding. Quality was categorized into 4 levels ranging from very low to high quality. In addition, we searched for evidence about values and preferences and cost of probiotic supplementation. We prepared the evidence-to-decision tables based on the estimates of the health effects, values and preferences and resource use.

During the meeting the guideline panel developed recommendations based on the evidence summaries and the evidence-to-decision tables. For each recommendation, the guideline panel considered and agreed on the following: the quality of the evidence, the balance of desirable and undesirable consequences of compared management options and the assumptions about the values and preferences associated with the decision. The guideline panel also explicitly took into account possible extent of resource use associated with alternative management options. Recommendations and their strength were decided by consensus and no recommendation required voting. The panel agreed on the final wording of recommendations and remarks with further qualifications for each recommendation. The final document including recommendations was reviewed and approved by all members of the guideline panel.

We labelled the recommendations as either “strong” or “conditional” according to the GRADE approach. We used the words “the panel members recommend” for strong recommendations and “suggest” for conditional recommendations. Table 1 provides suggested interpretation of strong and conditional recommendations by patients, clinicians and health care policy makers.

**Table 1:** **Interpretation of strong and conditional recommendations**

**Implications for:**	**Strong recommendation**	**Conditional recommendation**
Patients	Most individuals in this situation would want the recommended course of action, and only a small proportion would not.	The majority of individuals in this situation would want the suggested course of action, but many would not.
Clinicians	Most individuals should receive the intervention. Adherence to this recommendation according to the guideline could be used as a quality criterion or performance indicator. Formal decision aids are not likely to be needed to help individuals make decisions consistent with their values and preferences.	Recognize that different choices will be appropriate for individual patients and that you must help each patient arrive at a management decision consistent with his or her values and preferences. Decision aids may be useful in helping individuals to make decisions consistent with their values and preferences.
Policy makers	The recommendation can be adopted as policy in most situations.	Policymaking will require substantial debate and involvement of various stakeholders.

## Document review

A final draft document was reviewed by each member of the guideline panel, finalized, approved, and submitted to the WAO for peer review. The document was revised to incorporate the pertinent comments suggested by the external reviewers.

### How to use these guidelines

The WAO GLAD-P guidelines about the use of probiotics in the prevention of allergy in children are not intended to impose a standard of care. They provide the basis for rational decisions. Clinicians, patients, third-party payers, institutional review committees, other stakeholders, or the courts should never view these recommendations as dictates. No recommendation can take into account all of the often-compelling unique individual circumstances. Therefore, no one charged with evaluating health care professional’s actions should attempt to apply the recommendations from these guidelines by rote or in a blanket fashion.

Statements about the underlying values and preferences as well as qualifying remarks accompanying each recommendation are its integral parts and serve to facilitate more accurate interpretation. They should never be omitted when quoting or translating recommendations from these guidelines.

### Recommendations

**Question 1.** Should probiotics vs. no probiotics be used in pregnant women?

### Summary of the evidence

We found eight systematic reviews [15–22] that addressed this question. The review by Foolad [20] and Pelucchi [22] were the most recent and comprehensive. However, as with the other six reviews, they considered only the development of individual allergies as the main outcomes. For instance, the reviews of Foolad, [20] Betsi, [16] Doege, [17] Lee, [21] and Pelucchi [22] evaluated the development of atopic dermatitis in infancy, whereas Azad, [15] and Elazab [19] assessed the development of asthma/wheezing. The review by Dugoua [18] focused on the safety of probiotics administered during pregnancy. Thus, we could not rely on the information from those reviews alone.

We used the studies included in the systematic reviews and we systematically searched for additional studies of supplementation of probiotics in women during pregnancy. We identified 21 randomized controlled trials (RCTs) [23–43], of which only one [23] directly evaluated the use of probiotics given to women during their pregnancy only (direct evidence). In 4 of the remaining 20 studies probiotics were given to women during pregnancy and subsequently during breastfeeding, [24,25,27,28] in 8 studies probiotics were used during pregnancy and then in infants, [29–31,35–39] and in 8 studies probiotics were used in all of these populations [26,32–34,40–43]. We extracted data from the original publications and combined them in meta-analysis, if appropriate. These studies include probiotics given as supplements and as part of functional food. Owing to the heterogeneity of the interventions and limitations in reporting of original studies it was not possible to analyze the effects in each group separately. It was also not possible to analyze the effects of individual probiotic species and/or strains.

Follow-up in the studies ranged from 2 to 24 months after childbirth. Confidence in the estimated effects of probiotics on outcomes of interest was moderate to very low owing to the risk of bias, indirectness of the evidence and imprecision of the estimates (see evidence profile for question 1 in the Additional file 2).

Fifteen studies measured and reported development of eczema in the child [23–30,32,34,37–41]. The quality of the evidence for this outcome was moderate owing to a serious risk of bias. Although only one study [23] evaluated the intervention given to women exclusively during their pregnancy (direct evidence), we did not downgrade the quality of the evidence for indirectness since the direct and indirect bodies of evidence were congruent. The risk of eczema was reduced in children whose mothers received a probiotic during pregnancy, compared to placebo (risk ratio [RR] 0.72, 95% confidence interval [CI] 0.61 to 0.85).

Development of asthma/wheezing in the child was reported in 8 studies [23,24,29,35,36,40,42,43] and did not differ between the probiotic and placebo arms (RR 0.93, 95% CI of 0.76 to 1.15). Available evidence from 3 studies [27,29,33] does not indicate that probiotics given to pregnant women reduce the risk of developing food allergy in the child. However, there were very few events in these studies and thus the estimates are imprecise and confidence interval does not exclude an appreciable benefit or an appreciable harm (RR 1.49, 95% CI 0.58 to 3.81). Development of allergic rhinitis in the child was assessed in 5 studies [24,29,33,35,42]. No effect of probiotics on development of allergic rhinitis was observed (RR 0.86, 95% CI 0.44 to 1.7). Three studies reported development of “any allergy” [37,38,40]. No effect of probiotics was observed (RR 0.93, 95% CI 0.8 to 1.08).

#### Adverse effects

Although poorly documented and defined, most commonly reported adverse events were mild and short-term, i.e., vomiting, diarrhoea, rash, crying, and constipation. They were explicitly described in three individual studies [28,29,31] and no differences were detected between the probiotic and the placebo arms (RR 1.14, 95% CI 0.91 to 1.42). However, the confidence in this estimate is low due to indirectness of the evidence and risk of bias.

We found one health technology assessment (HTA) that evaluated the risk of adverse effects of probiotics in any population and age group [44]. It found 622 experimental and observational studies that primarily examined supplementation of Lactobacillus alone or in combination with other probiotics, often Bifidobacterium. Interventions and adverse events were poorly documented in the individual studies. In 235 studies only nonspecific safety statements were made (e.g. “well tolerated”). In this HTA, randomized trials failed to show an increased risk of any adverse event associated with short-term probiotic use (RR: 1.00, 95% CI: 0.93 to 1.07) as well as gastrointestinal, infectious or other adverse events, including serious adverse events (RR: 1.06, 95% CI: 0.97 to 1.16). However, the majority of studies included in this report explicitly excluded pregnant and breastfeeding women. Moreover, the long-term effects are unknown.

A small number of case reports described fungemia and bacteremia potentially associated with the administration of probiotics. Studies with a control group did not measure such events routinely. RCTs that included critically ill participants did not report an increased risk of adverse events.

### Desirable consequences

There is modest reduction of risk of developing eczema in children with probiotic supplementation in pregnant women (RR 0.72, 95% CI 0.61 to 0.85) when they are administered during the pregnancy only and/or during breastfeeding or to the infant.

### Undesirable consequences

Adverse effects were not different between the probiotic and placebo arms of the included studies. Based on the evidence from studies in pregnant women and all studies of probiotics included in the HTA report, panel members judged the risk of adverse effects to be low. The panel assumed that the burden of taking daily probiotic supplement is limited.

### Other considerations

We agreed that the values and preferences of women regarding the use of probiotics during pregnancy are likely to depend on cultural and socioeconomic background. Decision whether to use probiotics or not will also depend on women’s prior experience and whether they have already had children with allergy and the type of that allergy. The panel noted that some women with disorders of the immune system (e.g. autoimmune diseases) might not accept potential risks.

We explicitly considered the required resources. Prices of probiotics are likely to vary substantially depending on the setting, which may be a particularly important consideration in low and middle-income countries. A level and type of insurance may play a substantial role as well. We noted that from the perspective of health system probiotic supplementation in pregnancy might also be cost effective given that probiotics would be used for up to 9 months (in most studies probiotics were used during the last 3 month of pregnancy which, if applied, might also reduce the cost) and that the cost of treatment of eczema usually extends over many years. In some settings it may be prudent to consider equity as the access to probiotics may depend on socioeconomic status and coverage may depend on policymakers.

## Conclusions and research needs

The guideline panel determined that there is a likely net benefit from using probiotics in pregnant women. This recommendation is based on trials investigating single probiotics or mixtures of probiotics (See Additional file 3). We have not found differences in the effects among probiotics but that does not imply that such a difference does not exist. Similarly, further research of possible differences among the strains of the same species might be warranted. There is a need for development of instruments for evaluating the risk of allergy in children, since the family history predicts only about 30% of the population risk. There is also some evidence that first child is at higher risk for allergy than subsequent children. There is a need for rigorously designed and executed randomized trials of probiotics in pregnant women, especially those at higher risk for allergies in their children, that properly measure and report patient-important outcomes, including development of allergy, quality of life and adverse effects. Long-term follow-up of such studies to evaluate long-term effects is also needed. We noted the following additional research questions: 1) is the effect of natural probiotics in food different from that of supplementation and 2) is there an added benefit from probiotic supplementation in addition to natural probiotics? Future research, if done, may have an important impact on this recommendation.

## What others are saying

The European Academy of Allergy and Clinical Immunology (EAACI) Food Allergy and Anaphylaxis Guidelines state that “there is no evidence to recommend that women modify their diet during pregnancy or take any supplements such as probiotics in order to prevent food allergy in their children [45]”.

Guidelines from the US National Institute of Allergy and Infectious Diseases, [9] the European Society for Paediatric Gastroenterology, Hepatology, and Nutrition (ESPHGAN), [46] the World Gastroenterology Organisation Global Guidelines on Probiotics and Prebiotics [47], and the Food & Agriculture Organization (FAO)/World Health Organization (WHO) Guidelines on Probiotics [48] make no specific recommendations about the use of probiotics in pregnant women.

### Recommendation 1

***The WAO guideline panel suggests using probiotics in pregnant women at high risk for allergy in their children, because considering all critical outcomes, there is a net benefit resulting primarily from prevention of eczema****(conditional recommendation, very low quality evidence)*.

### Values and preferences

This recommendation places a relatively high value on prevention of eczema in children, and a relatively lower value on avoiding possible adverse effects.

### Explanations and other considerations

There is lack of evidence that probiotics prevent any other allergy. In most studies probiotics were used in the last 3 months of pregnancy. This recommendation applies to otherwise healthy women and cannot be generalized to those with compromised immune system function. High risk for allergy in a child is defined as biological parent or sibling with existing or history of allergic rhinitis, asthma, eczema, or food allergy.

**Question 2.** Should probiotics vs. no probiotics be used in breastfeeding mothers?

## Summary of the evidence

None of the systematic reviews that we identified directly evaluated using probiotics in breastfeeding mothers for the prevention of allergy in their children. Therefore, we performed a systematic review of randomized controlled trials of probiotics in breastfeeding mothers.

We found 13 RCTs that investigated the use of probiotics in breastfeeding mothers, [24–28,32–34,40–43,49] although only one evaluated probiotic supplementation exclusively during the breastfeeding period [49]. In the other 12 studies probiotics have also been given during pregnancy or to the infants. Follow-up in the studies ranged from 2 to 24 months after childbirth. Confidence in the estimated effects of probiotics on most outcomes of interest was very low owing to the risk of bias, indirectness of the evidence and imprecision of the estimates (see evidence profile for question 2 in the online Additional file 2).

Ten studies investigated the effect of probiotics given to breastfeeding mothers on development of eczema in infants [24–28,32,34,40,41,49]. Only one study [49] evaluated the intervention given to women exclusively during the breastfeeding period (direct evidence). However, we did not downgrade the quality of the evidence for indirectness since the direct and indirect bodies of evidence were congruent. The use of probiotics during the breastfeeding period reduced the rate of eczema in infants when compared to placebo (RR 0.61, 95% CI from 0.50 to 0.64). Asthma/wheezing was measured in 4 studies [24,40,42,43] in which the intervention was administered not only during the breastfeeding period but also during pregnancy and/or to the infant). No difference was observed between the probiotic and placebo arms but there were relatively few events and the confidence interval does not exclude an appreciable benefit or an appreciable harm (RR of 1.05, 95% CI from 0.59 to 1.87). Two studies assessed the risk of developing food allergy [27,33]. No difference was observed with probiotic supplementation (RR 1.7, 95% CI 0.58 to 4.96) but the confidence interval does not exclude an appreciable harm or an appreciable benefit. Three trials assessed development of allergic rhinitis [24,33,42]. Again, there were relatively few events and the results are very imprecise (RR 0.86, 95% CI 0.21 to 3.47. Two studies reported the risk of developing “any allergy” [40,49] and failed to show a benefit or harm from probiotic supplementation to breastfeeding mothers (RR 1.02, 95% CI 0.71 to 1.46).

### Adverse events

Adverse events were reported inconsistently. One randomized trial [28] with a mean follow-up of 24 months provided indirect and imprecise evidence of no difference between the probiotic and placebo arms (RR 1.52, 95% CI from 0.79 to 2.96). The aforementioned HTA report provided very low quality evidence about safety of probiotics in various populations and age groups [44]. However, the majority of studies included in this report explicitly excluded pregnant and breastfeeding women.

#### Desirable consequences

Probiotic supplementation to breastfeeding mothers reduced the risk of developing eczema in children (RR 0.61, 95% CI from 0.50 to 0.64). However, there is some concern about the directness of this evidence as in most studies probiotics were given not only during the breastfeeding period but also during pregnancy and/or to infants.

#### Undesirable consequences

Any estimate of potential adverse effects is very uncertain due to small number of patients and inadequate reporting. However, based on the evidence from all studies of probiotics, panel members judged the risk of adverse effects to be low. The panel assumed that the burden of taking daily probiotic supplement is limited.

#### Other considerations

We agreed that the considerations of values and preferences, resource implications and equity are likely similar to those in pregnant women. We also noted that the cost of probiotics is much lower than cost of a formula which may have an impact on the assessment of opportunity cost.

## Conclusions and research needs

The guideline panel determined that there is a likely net benefit from using probiotics in breastfeeding women. It is likely that probiotic supplementation in breastfeeding mothers reduces the risk of eczema in children. There is very low certainty that there is any effect of probiotic use by breastfeeding mothers on the development of other allergies in their children. We noted that, albeit possibly owing to chance, the relative magnitude of both benefits and downsides seems higher when probiotics were used by breastfeeding mothers compared to using them in pregnant women (see Additional file 2 – evidence profiles for questions 1 and 2).

We have not found differences in the effects among probiotics but that does not imply that such a difference does not exist. Similarly, further research of possible differences among the strains of the same species might be warranted. There is a need for rigorously designed and well-executed randomized trials of probiotics in breastfeeding women that would properly measure and report patient-important outcomes, including quality of life and adverse effects. Long-term follow-up of such studies to evaluate long-term effects is also needed. We noted the following additional research questions: 1) is the effect of natural probiotics in food different from that of supplementation and 2) is there an added benefit from probiotic supplementation in addition to natural probiotics? Future research, if done, may have an important impact on this recommendation.

## What others are saying

The EAACI Food Allergy and Anaphylaxis Guidelines state “there is no evidence to recommend that breastfeeding women should modify their diet or take any supplements such as probiotics in order to prevent food allergy in their children”. [45]. No other guideline that we reviewed made a specific recommendation about the use of probiotics in breastfeeding mothers [47,48].

### Recommendation 2

***The WAO guideline panel suggests using probiotics in women who breastfeed infants at high risk of developing allergy, because considering all critical outcomes, there is a net benefit resulting primarily from prevention of eczema****(conditional recommendation, very low quality evidence)*.

#### Values and preferences

This recommendation places a relatively high value on prevention of development of eczema, and relatively lower value on avoiding possible adverse effects.

#### Explanations and other considerations

There is lack of evidence that probiotics prevent any other allergy. This recommendation applies to otherwise healthy women and caution should be applied in generalizing to women with compromised immune system function. High risk for allergy in a child is defined as biological parent or sibling with existing or history of allergic rhinitis, asthma, eczema, or food allergy.

**Question 3.** Should probiotics vs. no probiotics be used in healthy **infants**?

### Summary of the evidence

We found 5 systematic reviews that assessed the use of probiotics in infants [15,19,50–52]. Most concentrated only on the evaluation of development of eczema (atopic dermatitis). Therefore, we performed a systematic review and we found 23 RCTs [26,29–43,53–59] in which probiotics were used in infants. Of these, 7 studies investigated the use of probiotics in infants only, [53–59] 8 studies assessed the use of probiotics in infants and in women during pregnancy, [29–31,35–39] and another 8 studies assessed the effect of probiotics given to pregnant women and subsequently breastfeeding mothers and infants [26,32–34,40–43]. We extracted data from the original publications and combined them in meta-analysis, if appropriate.

Follow-up in the included studies ranged from 4 to 36 months. The confidence in the estimated effects of probiotics on outcomes of interest was low to very low owing to the risk of bias, indirectness of the evidence and imprecision of the estimates. The only outcome for which the available evidence provides moderate confidence in the effect of probiotics is development of eczema (see evidence profile for question 3 in the Additional file 2).

Fifteen randomized trials measured development of eczema [26,29,30,32,34,37–41,54,55,57–59] Only 5 of the 10 studies provided direct evidence (i.e., evaluated the administration of probiotics only to infants); we did not downgrade the quality of the evidence because of indirectness since the direct and indirect bodies of evidence were congruent. Probiotics, when given to infants, decreased the risk of developing eczema compared to placebo (RR 0.81, 95% CI 0.70 to 0.94). Nine studies assessed development of asthma/wheezing 29,35,36,40,42,43,54,56,59]. No difference between the probiotic and placebo groups was observed (RR 0.98, 95% CI from 0.78 to 1.23). Only 3 of the 9 studies provided direct evidence but the direct and indirect bodies of evidence were congruent. Development of food allergy was measured in 5 trials [29,33,54–56] and no difference between the probiotics and placebo arms was noted (RR 0.9, 95% CI from 0.57 to 1.41). Four trials assessed development of allergic rhinitis [29,33,35,42]. These studies failed to demonstrate an effect of probiotics on development of allergic rhinitis in children (RR 0.83, 95% CI from 0.39 to 1.79). However, there was serious inconsistency in the results of the studies that we could not explain either by the risk of bias or by type of probiotic used or the population that received probiotics (pregnant women, breastfeeding mothers and/or infants). Four randomized trials [37,38,40,56] reported development of “any allergy”. No differences were detected between probiotic and placebo arms (RR 0.97, 95% CI from 0.85 to 1.12).

#### Adverse effects

Only 4 studies reported adverse events [29,31,53,55]. No differences were detected between the probiotic and placebo arms (RR 1.1, 95% CI 0.64 to 1.91). Two systematic reviews assessed the rate of adverse events from randomized trials and observational studies [50,51] showing similar results. The overview of systematic reviews by Foisy et al. [50] states that the only significant parent-reported event was more spitting up in their infants at one and two months of age (RR 1.88, 95% CI from 1.03 to 3.45; and RR 1.69, 95% CI from 1.02 to 2.80 respectively). The systematic review by Mugambi et al. [51] reported no significant effect of adding probiotics to infant formula on any adverse effects, including spitting up/regurgitation, gastrointestinal complaints, and diarrhoea. However, all estimates were imprecise owing to relatively low number of events.

#### Nutrition status

Three studies [31,53,58] assessed the nutrition status in infants, whether by measuring their body mass index, weight at the end of the follow-up, or change in weight. No difference was detected between intervention and control arms (standardized mean difference: 0.02 lower, 95% CI from 0.17 lower to 0.12 higher). One systematic review of probiotics added to infant formula investigated its impact on nutritional status and failed to find a difference between probiotic and placebo [51].

### Desirable consequences

Probiotic supplementation in infants is likely to reduce the risk of developing eczema.

### Undesirable consequences

Any estimate of potential adverse effects was graded as low certainty due to inadequate reporting in primary studies. However, given the available evidence, the guideline panel considered the risk of adverse effects most likely to be low. Panel members noted that certain preparations of probiotics might not be acceptable to some children because of unpleasant taste.

### Other considerations

If probiotics are used in infants, it is not clear when they should be started and how long they should be used. Also there is uncertainty about the dosage and whether the effectiveness differs for single probiotic strains compared with mixtures of several strains and/or species.

These considerations concern otherwise healthy infants in whom probiotics would be used for prevention of allergy. It does not concern using probiotics for specific indications in infants with specific medical condition, e.g. for reducing the risk of antibiotic-induced diarrhea or prevention of necrotizing enterocolitis in premature infants [50].

## Conclusions and research needs

The guideline panel determined that there is a likely net benefit from using probiotics in infants. It is likely that probiotic supplementation in infants reduces the risk of developing eczema. There is very low certainty that there is any effect of probiotics on other outcomes. We have not found differences in the effects among probiotics but that does not imply that such a difference does not exist.

There is a need for rigorously designed and well executed randomized trials of probiotics in infants that would measure and adequately report patient-important outcomes, including adverse effects. Similarly, further research of possible differences among the strains of the same species might be warranted. We noted the following additional research questions: 1) is the effect of natural probiotics in food different from that of supplementation and 2) is there an added benefit from probiotic supplementation in addition to natural probiotics? Future research, if done, may have an important impact on this recommendation.

### What others are saying

The EAACI Food Allergy and Anaphylaxis Guidelines state that “there is no evidence to recommend prebiotics or probiotics or other dietary supplements based on particular nutrients to prevent food allergy”. [45] The World Gastroenterology Organization Global Guidelines establish that “the strongest evidence is for the prevention of atopic dermatitis when certain probiotics are administered to pregnant mothers and newborns up to 6 months of age [47]”. The FAO/WHO guidelines do not give specific recommendations about the use of probiotics in infants [48].

### Recommendation 3

***The WAO guideline panel suggests using probiotics in infants at high risk of developing allergies, because considering all critical outcomes, there is a net benefit resulting primarily from prevention of eczema****(conditional recommendation, very low quality evidence)*.

#### Values and preferences

This recommendation places a relatively high value on prevention of eczema, and relatively lower value on avoiding possible adverse effects.

#### Explanations and other considerations

There is lack of evidence that probiotics prevent any other allergy. This recommendation applies to otherwise healthy infants with the goal of preventing allergies. It does not apply to the use of probiotics for other indications, e.g. prevention of antibiotic-associated diarrhea or enterocolitis in premature infants.

### Priorities for revision of the guidelines

#### Plans for updating these guidelines

Guidelines are living documents. To remain useful, they need to be updated regularly as new information accumulates. A revision of this document will be needed, because there was limited evidence for many clinical questions. This document will be updated when major new research is published. The need for update will be determined not later than in 2018.

#### Updating or adapting recommendations locally

The methods used to develop these guidelines are transparent. The recommendations have been developed to be as specific and detailed as possible without losing sight of the simplicity of the document. Since GLAD-P are meant as international guidelines, the guideline panel encourages feedback on its all aspects including their applicability in individual countries. This feedback will be considered when revising the document.

Adaptation of these guidelines will be necessary in many circumstances. Depending on when such a process takes place, the following steps should be taken:

- ◾ Appointing a guideline committee comprising clinicians and methodologists
- ◾ Determining the scope of the localized guidelines
- ◾ Defining the clinical questions to be addressed
- ◾ Updating the evidence profiles and evidence-to-decision tables, if necessary
- ◾ Reviewing the recommendations in the GLAD-P guidelines (the recommendations may need to be modified at a local level, depending on the local values and preferences, availability of medications, costs, etc.)
- ◾ Disseminating the guidelines, with a clear “use by” date
- ◾ Developing a method to obtain feedback and plans for review and update.

### Priorities for research

During the guideline development process we identified a need for more data on specific topics. This results in the following recommendations for research. We summarize these gaps in the evidence as research recommendations, to assist those in a position to provide such information by the design and execution of specific research projects.

Specific research needs to be addressed:

1. Development of instruments for evaluating the risk of allergy in children (the family history predicts only about 30% of the population risk).
2. Evaluation of effects of using probiotics in formulas.
3. Evaluation of effects of using probiotics in breastfeeding mothers specifically in that period (as opposed to intervention administered also during pregnancy and to children).
4. Evaluation of the effects of different ways of administration of probiotics , e.g. as milk or dairy supplements, stand-alone supplements, etc.
5. Performance of rigorously designed, adequately powered, and well executed randomized trials of probiotics in infants who did not receive probiotics prenatally and/or during breastfeeding; studies should include infants considered to be at high and low/average risk for allergies and should properly report patient-important outcomes, including adverse effects. The estimated optimal information size for this question is from approximately 2500 participants (for eczema) to 27,000 participants (for food allergy). However, for the evaluation of adverse effects a large compilation of RCTs as well as observational studies might be necessary with possible thousands of observations.
6. Evaluation which of the 3 populations (pregnant women, breastfeeding mothers and infants) should receive probiotics – whether there is a larger benefit with supplementation in one or combination of these populations and, if so, which populations to target.
7. Evaluation whether any effect of probiotics is a class effect or differs among species and strains of microorganisms. An effect of shelf life on effectiveness of probiotics also warrants further investigation [60].

## Additional files

Additional file 1:
**Declaration of potential conflicts of interest (within last 4 years).**
Additional file 2:
**Evidence profiles.**
Additional file 3:
**Probiotic strains and dosages used in the included studies.**
