# Supplementary material for: World Allergy Organization-McMaster University Guidelines for Allergic Disease Prevention (GLAD-P): Probiotics
Source: World Allergy Organ J. 2015 Jan 27;8(1):4. doi: 10.1186/s40413-015-0055-2 (PMC4307749; doi:10.1186/s40413-015-0055-2)
Supplement: Additional file 1: — Declaration of potential conflicts of interest (within last 4 years). [file 40413_2015_55_MOESM1_ESM.docx]

## Additional file 1. Declaration of potential conflicts of interest (within last 4 years)

| Panel member | Declaration |
| --- | --- |
| Kangmo Ahn | Declares no conflicts of interest related to this guideline |
| Suleiman Al-Hammadi | Received honoraria for speakers bureau, giving talks, and sponsorship at meetings from Danone Nutrition. Received honoraria from MSD for speaking. |
| Kirsten Beyer | Consult as technical advisor for DuPont, Unilever and Danone |
| Jan Brożek | Received research support from WAO for development of DRACMA and GLAD-P guidelines |
| Wesley Burks | Current consulting agreements with: Dynavax Technologies Corp., Genalyte, GLG Research, Perrigo Company, Regeneron Pharmaceuticals, NIH Grant support c. Abbott Laboratories, Levine's Children's Hospital, Mylan Speciality, Perosphere, Inc. Past consulting agreements: ActoGeniX, Curalogic, Dow AgroSciences, ExploraMed Development, McNeill Nutritionals, Merck, Novartis Pharma AG, Sanofi-Aventis US, Schering Plough, Unilver |
| Giorgio Walter Canonica | Declares no conflicts of interest related to this guideline |
| Carlos Cuello-García | Declares no conflicts of interest related to this guideline |
| Motohiro Ebisawa | Declares no conflicts of interest related to this guideline |
| Alessandro Fiocchi | Research support from Danone. Has received travel support for meetings for presentation of DRACMA guidelines in South East Asia from Danone Malaysia. Support for travel and honorarium for speaking from Ordesa Group. |
| Rose Kamenwa | Declares no conflicts of interest related to this guideline |
| Bee Wah Lee | Received research support from Abbott Nutrition, Danone Nutrition, Nestle Nutrition Honoraria for speakers bureau, giving talks, sponsorship at meetings |
| Haiqi Li | Declares no conflicts of interest related to this guideline |
| Ruby Pawankar | Received support from Danone Asia Pacific for act as a speaker at a Food allergy Asia Pacific symposium 2012 |
| Susan Prescott | Advisory Board - Nestle Nutrition Institute (Australasia), Advisory Board for Danone (Asia Pacific), travel grant as Invited Speaker - ALK Abello, Travelling Fellowship for Russian Doctor to visit her laboratory |
| John Riva | Declares no conflicts of interest related to this guideline |
| Lanny Rosenwasser | Research Grant from Novartis /Genentech Roche (finished in 2011). Advisory board and speakers board for Astra Zeneca and Novartis/Genentech (last applicable in 2012), 5 patents on the biology of IL-1 and it's uses. |
| Hugh Sampson | Consultant– Danone Research for Specialized Nutrition Scientific Advisory Committee. Research support from the NIAID and NIH |
| Holger Schünemann | Received research support from WAO for development of DRACMA and GLAD-P guidelines |
| Michael D. Spigler | Declares no conflicts of interest related to this guideline |
| Luigi Terracciano | Heinz-Plada Italy Medical consultant for website. Travel support from World Allergy Organization. |
| Andrea Vereda-Ortiz | EAACI paid travel expenses for attendance to the meeting in Milan last June (congress registration, plane and hotel).  Worked until November 2013 for a pharmaceutical company (Almirall) as a medical advisor. Worked for Almirall between October 2009 and September 2013. Previously worked in Stallergenes corporation (specific immunotherapy). |
| Susan Waserman | Declares no conflicts of interest related to this guideline |
| Juan José Yepes Nuñez | Declares no conflicts of interest related to this guideline |
