# Supplementary material for: World Allergy Organization-McMaster University Guidelines for Allergic Disease Prevention (GLAD-P): Probiotics
Source: World Allergy Organ J. 2015 Jan 27;8(1):4. doi: 10.1186/s40413-015-0055-2 (PMC4307749; doi:10.1186/s40413-015-0055-2)
Supplement: Additional file 2: — Evidence profiles. [file 40413_2015_55_MOESM2_ESM.docx]

**Additional file 2. Evidence profiles**

EVIDENCE PROFILE (GRADE) – QUESTION 1

**Question**: Should probiotics vs. no probiotics be used in pregnant women for prevention of allergy in their children?

**Author(s)**: Jan Brozek, Carlos Cuello, Juan J. Yepes, Holger Schünemann

**Bibliography (systematic reviews)**: World Allergy Organisation systematic review

| **Quality assessment** | | | | | | | **№ of patients** | | **Effect** | | **Quality** | **Importance** |
| --- | --- | --- | --- | --- | --- | --- | --- | --- | --- | --- | --- | --- |
| **№ of studies** | **Study design** | **Risk of bias** | **Inconsistency** | **Indirectness** | **Imprecision** | **Other considerations** | **probiotics** | **no probiotics** | **Relative**  **(95% CI)** | **Absolute**  **(95% CI)** |  |  |
| Eczema in a child (follow up: range 12 to 24 months) | | | | | | | | | | | | |
| 15 | randomised trials | serious ^1^ | not serious | not serious ^2^ | not serious | none | 426/1835 (23.2%) | 527/1674 (31.5%) | **RR 0.72**  (0.61 to 0.85) | 88 fewer per 1000 (from 47 fewer to 123 fewer) | ⨁⨁⨁◯  MODERATE | CRITICAL |
| Asthma/wheezing in a child (follow up: range 12 to 24 months) | | | | | | | | | | | | |
| 8 | randomised trials | serious ^3^ | not serious | not serious ^2^ | not serious | none | 156/1118 (14.0%) | 168/1135 (14.8%) | **RR 0.93**  (0.76 to 1.15) | 10 fewer per 1000 (from 22 more to 36 fewer) | ⨁⨁⨁◯  MODERATE | CRITICAL |
| Food allergy in a child (follow up: range 12 to 24 months) | | | | | | | | | | | | |
| 3 | randomised trials | serious ^8^ | not serious | serious ^4^ | serious ^5^ | none | 10/177 (5.6%) | 7/178 (3.9%) | **RR 1.49**  (0.58 to 3.81) | 19 more per 1000 (from 17 fewer to 111 more) | ⨁◯◯◯  VERY LOW | CRITICAL |
| Allergic rhinitis in a child (follow up: range 12 to 24 months) | | | | | | | | | | | | |
| 5 | randomised trials | serious ^6^ | not serious | serious ^7^ | serious ^5^ | none | 108/867 (12.5%) | 108/876 (12.3%) | **RR 0.86**  (0.44 to 1.7) | 17 fewer per 1000 (from 69 fewer to 86 more) | ⨁◯◯◯  VERY LOW | CRITICAL |
| Any allergy in a child (follow up: range 12 to 24 months) | | | | | | | | | | | | |
| 3 | randomised trials | serious ^9^ | not serious | serious ^7^ | not serious | none | 198/578 (34.3%) | 212/574 (36.9%) | **RR 0.93**  (0.8 to 1.08) | 26 fewer per 1000 (from 30 more to 74 fewer) | ⨁⨁◯◯  LOW | CRITICAL |
| Adverse events in a child (follow up: range 12 to 24 months) | | | | | | | | | | | | |
| 3 | randomised trials | serious ^10^ | not serious | serious ^7^ | not serious | none | 101/394 (25.6%) | 88/397 (22.2%) | **RR 1.14**  (0.91 to 1.42) | 31 more per 1000 (from 20 fewer to 93 more) | ⨁⨁◯◯  LOW | CRITICAL |

MD – mean difference, RR – relative risk

1. Of the 15 included studies, an unclear risk of bias for allocation concealment was present and 10, incomplete outcome data (attrition bias) in 11 studies, and in 4 there was an unclear random sequence generation and unclear blinding of assessment. Only two studies were at low risk of bias for all items
2. Although only one study evaluated the intervention given to women during their pregnancy only (direct evidence), we did not downgrade for indirectness since the direct and indirect bodies of evidence were similar and congruent.
3. Of the 8 included studies, unclear risk of bias for allocation concealment was present in 5, incomplete outcome data (attrition bias) in 7, and an unclear random sequence generation process and blinding of assessment was present in one study each.
4. In all three studies the intervention was given to women during their pregnancy plus during breastfeeding and/or to their infants, hence considering this an indirect body of evidence
5. Confidence interval is wide and does not exclude appreciable benefit or harm
6. Four studies with unclear risk of bias on concealment allocation and incomplete outcome data (attrition bias). Only one study with low risk of bias in all items
7. In all studies the intervention was given to women during their pregnancy plus during breastfeeding and/or to their infants, hence considering this an indirect body of evidence
8. Unclear risk of bias for incomplete outcome data was present in the three studies. One study presented an unclear risk of bias in the allocation concealment process.
9. All three studies with unclear risk of bias for allocation concealment and incomplete outcome assessment
10. Two studies with unclear risk of bias for allocation concealment, blinding of patients and incomplete outcome data assessment.

EVIDENCE PROFILE (GRADE) – QUESTION 2

**Question**: Should probiotics vs. no probiotics be used in breastfeeding mothers for prevention of allergy in their children?

**Author(s)**: Jan Brozek, Carlos Cuello, Juan J. Yepes, Holger Schünemann

**Bibliography (systematic reviews)**: World Allergy Organisation systematic review

| **Quality assessment** | | | | | | | **№ of patients** | | **Effect** | | **Quality** | **Importance** |
| --- | --- | --- | --- | --- | --- | --- | --- | --- | --- | --- | --- | --- |
| **№ of studies** | **Study design** | **Risk of bias** | **Inconsistency** | **Indirectness** | **Imprecision** | **Other considerations** | **probiotics** | **no probiotics** | **Relative**  **(95% CI)** | **Absolute**  **(95% CI)** |  |  |
| Eczema in a child (follow up: range 12 to 24 months) | | | | | | | | | | | | |
| 10 | randomised trials | serious ^1^ | not serious | not serious ^2^ | not serious | none | 190/888 (21.4%) | 256/707 (36.2%) | **RR 0.61**  (0.5 to 0.74) | 141 fewer per 1000 (from 94 fewer to 181 fewer) | ⨁⨁⨁◯  MODERATE | CRITICAL |
| Asthma / wheezing – Breast-feeding women only (follow up: range 12 to 24 months) | | | | | | | | | | | | |
| 4 | randomised trials | serious ^3^ | not serious | serious ^4^ | serious ^5^ | none | 48/392 (12.2%) | 51/409 (12.5%) | **RR 1.05**  (0.59 to 1.87) | 6 more per 1000 (from 51 fewer to 108 more) | ⨁◯◯◯  VERY LOW | CRITICAL |
| Food allergy – Breast-feeding women only (follow up: range 12 to 24 months) | | | | | | | | | | | | |
| 2 | randomised trials | serious ^6^ | not serious | serious ^7^ | serious ^5^ | none | 8/82 (9.8%) | 5/85 (5.9%) | **RR 1.7**  (0.58 to 4.96) | 41 more per 1000 (from 25 fewer to 233 more) | ⨁◯◯◯  VERY LOW | CRITICAL |
| Allergic rhinitis – Breast-feeding women only (follow up: range 12 to 24 months) | | | | | | | | | | | | |
| 3 | randomised trials | serious ^8^ | serious ^9^ | serious ^10^ | serious ^5^ | none | 15/327 (4.6%) | 19/337 (5.6%) | **RR 0.86**  (0.21 to 3.47) | 8 fewer per 1000 (from 45 fewer to 139 more) | ⨁◯◯◯  VERY LOW | CRITICAL |
| Any allergy – Breast-feeding women only (follow up: range 12 to 24 months) | | | | | | | | | | | | |
| 2 | randomised trials | serious ^11^ | not serious | serious ^7^ | serious ^5^ | none | 31/65 (47.7%) | 30/64 (46.9%) | **RR 1.02**  (0.71 to 1.46) | 9 more per 1000 (from 136 fewer to 216 more) | ⨁◯◯◯  VERY LOW | CRITICAL |
| Adverse events (follow up: mean 24 months) | | | | | | | | | | | | |
| 1 | randomised trial | not serious | not serious | serious ^12^ | serious ^5^ | none | 35/79 (44.3%) | 24/70 (34.3%) | **RR 1.52**  (0.79 to 2.96) | 178 more per 1000 (from 72 fewer to 672 more) | ⨁⨁◯◯  LOW | CRITICAL |

MD – mean difference, RR – relative risk

1. Unclear risk of bias for incomplete outcome data in 9 studies, allocation concealment in 5, random sequence generation in 3, and blinding of participants in 2. There was also an unclear risk of bias for blinding of outcome assessment in one study.
2. Although only one study evaluated the intervention given to women during the breastfeeding period only (direct evidence), we did not downgrade for indirectness since the direct and indirect bodies of evidence were similar and congruent.
3. The 4 included studies had an unclear risk of bias for incomplete outcome assessment (attrition bias), two had an unclear description of the allocation concealment process and one did not describe the random sequence generation
4. In the four included studies the intervention was administered either during the pregnancy and breastfeeding periods, during breastfeeding and to the infant, or during the three periods (pregnancy, breastfeeding and to the infant)
5. Wide confidence interval which does not exclude appreciable benefit or harm
6. Both studies with unclear risk of bias for incomplete outcome assessment (attrition bias)
7. In the two included studies the intervention was administered either during the pregnancy and breastfeeding periods, during breastfeeding and to the infant, or during the three periods (pregnancy, breastfeeding and to the infant)
8. Random sequence generation and allocation concealment (selection bias) were not clearly described in one study. In the three included studies outcome data was considered incomplete (attrition bias).
9. Statistical heterogeneity between the included studies
10. In the three included studies the intervention was administered either during the pregnancy and breastfeeding periods, during breastfeeding and to the infant, or during the three periods (pregnancy, breastfeeding and to the infant)
11. Both studies with incomplete outcome data (attrition bias) and unclear description of allocation concealment (selection bias)
12. In the included study the intervention was administered during pregnancy and breastfeeding periods

EVIDENCE PROFILE (GRADE) – QUESTION 3

**Question**: Should probiotics vs. no probiotics be used in infants for prevention of allergies?

**Author(s)**: Jan Brozek, Carlos Cuello, Juan J. Yepes, Holger Schünemann

**Bibliography (systematic reviews)**: World Allergy Organisation systematic review

| **Quality assessment** | | | | | | | **№ of patients** | | **Effect** | | **Quality** | **Importance** |
| --- | --- | --- | --- | --- | --- | --- | --- | --- | --- | --- | --- | --- |
| **№ of studies** | **Study design** | **Risk of bias** | **Inconsistency** | **Indirectness** | **Imprecision** | **Other considerations** | **probiotics** | **no probiotics** | **Relative**  **(95% CI)** | **Absolute**  **(95% CI)** |  |  |
| Eczema in an infant (follow up: range 6 to 24 months) | | | | | | | | | | | | |
| 15 | randomised trials | serious ^1^ | not serious | not serious ^2^ | not serious | none | 423/1798 (23.5%) | 479/1649 (29.0%) | **RR 0.81**  (0.7 to 0.94) | 55 fewer per 1000 (from 17 fewer to 87 fewer) | ⨁⨁⨁◯  MODERATE | CRITICAL |
| Asthma / wheezing in an infant (follow up: range 6 to 36 months) | | | | | | | | | | | | |
| 9 | randomised trials | serious ^3^ | not serious | serious ^4^ | not serious | none | 146/1064 (13.7%) | 152/1081 (14.1%) | **RR 0.98**  (0.78 to 1.23) | 3 fewer per 1000 (from 31 fewer to 32 more) | ⨁⨁◯◯  LOW | CRITICAL |
| Food allergy in an infant (follow up: range 6 to 24 months) | | | | | | | | | | | | |
| 5 | randomised trials | serious ^5^ | not serious | serious ^6^ | serious ^7^ | none | 29/323 (9.0%) | 33/321 (10.3%) | **RR 0.9**  (0.57 to 1.41) | 10 fewer per 1000 (from 42 more to 44 fewer) | ⨁◯◯◯  VERY LOW | CRITICAL |
| Allergic rhinitis in an infant (follow up: range 12 to 36 months) | | | | | | | | | | | | |
| 4 | randomised trials | serious ^8^ | serious ^9^ | serious ^10^ | serious ^7^ | none | 107/729 (14.7%) | 107/736 (14.5%) | **RR 0.83**  (0.39 to 1.79) | 25 fewer per 1000 (from 89 fewer to 115 more) | ⨁◯◯◯  VERY LOW | CRITICAL |
| Any allergy in an infant (follow up: range 6 to 24 months) | | | | | | | | | | | | |
| 4 | randomised trials | serious ^11^ | not serious | serious ^12^ | not serious | none | 239/655 (36.5%) | 246/650 (37.8%) | **RR 0.97**  (0.85 to 1.12) | 11 fewer per 1000 (from 45 more to 57 fewer) | ⨁⨁◯◯  LOW | CRITICAL |
| Adverse events in infant (follow up: range 6 to 36 months) | | | | | | | | | | | | |
| 4 | randomised trials | serious ^13^ | not serious | serious ^14^ | serious ^7^ | none | 118/408 (28.9%) | 114/421 (27.1%) | **RR 1.1**  (0.64 to 1.91) | 27 more per 1000 (from 97 fewer to 246 more) | ⨁◯◯◯  VERY LOW | CRITICAL |
| Nutritional status in infant (follow up: range 3 to 36 months; assessed with: growth and weight) | | | | | | | | | | | | |
| 3 | randomised trials | serious ^15^ | not serious | serious ^16^ | serious ^7^ | none | 348 | 360 | – | SMD **0.02 lower**  (0.17 lower to 0.12 higher) | ⨁◯◯◯  VERY LOW | CRITICAL |

MD – mean difference, RR – relative risk

1. In seven studies there was an unclear or incomplete assessment of outcome data (attrition bias). In six there was an unclear description of the allocation concealment process. In four studies the random sequence generation was not explicitly described. The blinding of outcome assessment and the blinding of participants were not described in detail in two and one study respectively.
2. Although five of the ten studies were considered direct, i.e., evaluated the administration of probiotics only to infants and not to the mother during pregnancy or breastfeeding period, we did not downgrade for indirectness since the direct and indirect bodies of evidence were similar and congruent.
3. In six studies there was an unclear risk of bias for allocation concealment whereas in four the random sequence generation was not described (selection bias). One study was not blinded and two did not describe the blinding of the assessment (performance and detection bias). Seven studies had an incomplete outcome assessment (attrition bias).
4. Although three of the nine studies were considered direct, i.e., evaluated the administration of probiotics only to infants and not to the mother during pregnancy or breastfeeding period, we did not downgrade for indirectness since the direct and indirect bodies of evidence were similar and congruent.
5. In four studies there was an incomplete or unclear assessment of outcome data (attrition bias). In three studies the allocation concealment was not described and the random sequence generation, blinding of participants and assessors were not described in one study each.
6. Three of the five included studies provided direct evidence, i.e., evaluated the administration of probiotics only to infants and not to the mother during pregnancy or breastfeeding period; we did not downgrade for indirectness since the direct and indirect bodies of evidence were similar and congruent.
7. Wide confidence interval which does not exclude appreciable benefit or harm.
8. The four studies had an incomplete assessment of outcome data. Two did not describe in detail the allocation concealment.
9. The four studies were heterogeneous in their results (statistical heterogeneity) mainly explained by one study (Wickens 2012).
10. The four studies provided indirect evidence, i.e., evaluated the administration of probiotics to infants plus to the mother during pregnancy, or to infants plus to the mother during the breastfeeding period or both situations.
11. The allocation concealment process was not described in three studies. Outcome data was incomplete or unclear in three studies. The blinding of participants was not detailed in two studies. The blinding of outcome assessment was not described in one study.
12. Three of the four studies provided indirect evidence, i.e., evaluated the administration of probiotics to infants plus to the mother during pregnancy, or to infants plus to the mother during the breastfeeding period or both situations.
13. In three studies there was an unclear outcome data assessment (attrition bias) and an incomplete or absent description of the allocation concealment method. In two studies there was no description of the blinding of participants. One study each did not describe the random sequence generation and the blinding of the outcome assessment.
14. Two of the four studies provided indirect evidence, i.e., evaluated the administration of probiotics to infants plus to the mother during pregnancy, or to infants plus to the mother during the breastfeeding period or both situations.
15. Allocation concealment, blinding of participants and incomplete outcome data assessment was unclear in two studies each. Random sequence generation and blinding of outcome assessment was unclear in one study each.
16. One of the three studies provided indirect evidence, i.e., evaluated the administration of probiotics to infants plus to the mother during pregnancy, or to infants plus to the mother during the breastfeeding period or both situations.
