# Supplementary material for: World Allergy Organization-McMaster University Guidelines for Allergic Disease Prevention (GLAD-P): Probiotics
Source: World Allergy Organ J. 2015 Jan 27;8(1):4. doi: 10.1186/s40413-015-0055-2 (PMC4307749; doi:10.1186/s40413-015-0055-2)
Supplement: Additional file 3: — Probiotic strains and dosages used in the included studies. [file 40413_2015_55_MOESM3_ESM.docx]

## Additional file 3. Probiotic strains and dosages used in the included studies

| Study | Probiotic(s) administered as intervention and compared to placebo or no probiotics |
| --- | --- |
| Abrahamsson et al. 2007 | Lactobacillus reuteri ATCC 55730 (1x108 CFU) daily from gestational week 36 until delivery. |
| Allen et al. 2010 | Lactobacillus salivarius CUL61; Lactobacillus paracasei CUL08; Bifidobacterium animalis subsp. lactis CUL34; Bifidobacterium bifidum CUL20, with a total of 1 x 10^10^ CFUs were administered daily to women during the last month of pregnancy and to infants aged 0–6 months |
| Allen et al. 2012 | Lactobacillus salivarius CUL61; Lactobacillus paracasei CUL08; Bifidobacterium animalis subsp. lactis CUL34; Bifidobacterium bifidum CUL20, with a total of 1 x 10^10^ CFUs |
| Boyle et al. 2011 | Lactobacillus rhamnosus GG (LGG) 1.8 x 10^10^ CFU/day) from 36 weeks gestation until delivery |
| Dotterud et al. 2010 | Lactobacillus rhamnosus GG (LGG), Bifidobacterium animalis subsp. lactis Bb-12 (Bb-12), Lactobacillus acidophilus La-5 (La-5), equalling 5 x 10^10^ CFU of LGG and Bb-12, and 5 x 10^9^ of La-5 per day. |
| Hascoet et al. 2011 | Bifidobacterium longum BL999 in formula with 2 x10^7^ CFU/g |
| Huurre et al. 2008 | Lactobacillus rhamnosus GG (LGG), Bifidobacterium lactis Bb12 (Bb-12) 1 x 10^10^ CFU/day each. |
| Kalliomaki et al. 2001 | Lactobacillus rhamnosus GG (LGG) ATCC 53103, 1 x 10^10^ CFU daily for 2 to 4 weeks before delivery. |
| Kalliomaki et al. 2003 | Lactobacillus rhamnosus GG (LGG) ATCC 53103, 1 x 10^10^ CFU daily for 2 to 4 weeks before delivery. |
| Kalliomaki et al. 2007 | Lactobacillus rhamnosus GG (LGG) ATCC 53103, 1 x 10^10^ CFU daily for 2 to 4 weeks before delivery. |
| Kim et al. 2010 | Bifidobacterium bifidum BGN4, Bifidobacterium lactis AD011, and Lactobacillus acidophilus AD031, each at a dose of 1.6 x 10^9^ CFU once daily from 8 weeks before the expected delivery to 3 months after delivery. Infants were fed the same powder dissolved in breast milk, infant formula, or sterile water from 4 to 6 months of age. |
| Kopp et al. 2008 | Lactobacillus GG (American Type Culture Collection 53103); 5x10^9^ CFU twice daily for 4 to 6 weeks before expected delivery. |
| Kuitunen et al. 2009 | Lactobacillus rhamnosus GG (LGG) (5x10^9^ CFU), Lactobacillus rhamnosus LC705 (DSM 7061) (5x10^9^ CFU), Bifidobacterium breve Bb99 (DSM 13692) (2x10^8^ CFU), Propionibacterium freudenreichii ssp. shermanii JS (DSM 7076) (2x10^9^ CFU) twice daily, from 36 weeks of gestation. Infants continued the mix of probiotics once daily until 6 months of age. |
| Kukkonen et al. 2007 | Lactobacillus rhamnosus GG (LGG) (5x10^9^ CFU), Lactobacillus rhamnosus LC705 (DSM 7061) (5x10^9^ CFU), Bifidobacterium breve Bb99 (DSM 13692) (2x10^8^ CFU), Propionibacterium freudenreichii ssp. shermanii JS (DSM 7076) (2x10^9^ CFU) twice daily, from 36 weeks of gestation. Infants continued the mix of probiotics once daily until 6 months of age. |
| Kukkonen et al. 2011a | Lactobacillus rhamnosus GG (LGG) (5x10^9^ CFU), Lactobacillus rhamnosus LC705 (DSM 7061) (5x10^9^ CFU), Bifidobacterium breve Bb99 (DSM 13692) (2x10^8^ CFU), Propionibacterium freudenreichii ssp. shermanii JS (DSM 7076) (2x10^9^ CFU) twice daily, from 36 weeks of gestation. Infants continued the mix of probiotics once daily until 6 months of age |
| Lodinova-Zadnikova et al. 2010 | Escherichia coli probiotic (Colinfant) (0.8 x10^9^ CFU) within 48 hours after birth and 3 times weekly thereafter for 4 weeks. |
| Marschan et al. 2008 | Lactobacillus rhamnosus GG (ATCC 53103) 5 x109 CFU, L. rhamnosus LC705 5 x109CFU, Bifidobacterium breve Bb99 2 x108CFU, and Propionibacterium freudenreichii ssp. Shermanii JS 2 x109CFU twice a day for 2 to 4 weeks before delivery; infants during the first 6 months of life. |
| Morisset et al. 2011 | Formula with Bifidobacterium breve C50 and Streptococcus thermophilus 065 (HKBBST) – CFU dosage not mentioned |
| Niers et al. 2009 | Bifidobacterium bifidum W23, Bifidobacterium lactis W52 (bifidobacterium infantis), and Lactococcus lactis W58, once daily 3 x10^9^ CFU; (or 1 x10^9^ CFU of each strain)  to the pregnant mothers during the last 6 weeks of pregnancy and postnatally for 12 months to their offspring. |
| Ortiz-Andrellucchi et al. 2008 | Milk (administered to the breastfeeding mothers) fermented with Lactobacillus casei DN 114001 after delivery, 3 times per day for 4 weeks. Dosage not mentioned. |
| Ou et al. 2012 | Lactobacillus GG; ATCC 53103; 1 x10^10^ CFU daily from the second trimester of pregnancy |
| Prescott et al. 2008 | Lactobacillus acidophilus (3x10^9^ CFU) within 48 hours of delivery, once a day for 6 months. |
| Rautava et al. 2002 | Lactobacillus rhamnosus GG (LGG) ATCC 53103; daily dose, 2 × 10^10^ during the 4 weeks before delivery and during breastfeeding. |
| Rautava et al. 2012 | Lactobacillus rhamnosus LPR (CGMCC 1.3724) and Bifidobacterium longum BL999 (ATCC: BAA-999) or the combination ST11 and BL999 (ST111BL999) consisting of L paracasei ST11 (CNCM 1-2116) and B longum BL999. Daily dose for each probiotic was 1 x10^9^ CFU, 2 months before the expected day of delivery and continued during breast-feeding until the child was 2 months of age. |
| Soh et al. 2009 | Milk formula (administered to infants) with Bifidobacterium longum BL999 1 x10^7^ CFU, and Lactobacillus rhamnosus (LPR) 2 x10^7^ CFU daily for the first six months of life. |
| Taylor et al. 2007 | Lactobacillus acidophilus LAVRI-A1 administered to infants (3x10^9^ CFU) once a day until the 6 months of life. |
| West et al. 2009 | Cereal supplemented with LF19 (Lactobacillus paracasei ssp. paracasei strain F19) daily from 4 to 13 months of life. One serving contained 1 x10^8^ CFU |
| Wickens et al. 2008 | Lactobacillus rhamnosus HN001 6 x10^9^ CFU, B animalis subsp lactis HN019 (9 x10^9^ CFU/d);  both once daily, from 35 weeks of gestation, during breastfeeding, and to the infant up to 24 months of life. |
| Wickens et al. 2012 | Lactobacillus rhamnosus HN001 6 x10^9^ CFU, B animalis subsp lactis HN019 (9 x10^9^ CFU/d);  both once daily, from 35 weeks of gestation, during breastfeeding, and to the infant up to 24 months of life. |

CFU, Colony-Forming Unit
